# Therapeutic Potential of Myricitrin in a *db*/*db* Mouse Model of Type 2 Diabetes

**DOI:** 10.3390/molecules30071460

**Published:** 2025-03-25

**Authors:** Sang Ryong Kim, Young-Je Kim, HwiCheol Kim, Sojeong Park, Un Ju Jung

**Affiliations:** 1 School of Life Science and Biotechnology, BK21 FOUR KNU Creative BioResearch Group, Kyungpook National University, 80 Daehak-ro, Buk-gu, Daegu 41566, Republic of Korea; srk75@knu.ac.kr; 2 Department of Food Science and Nutrition, Kyungpook National University, 80 Daehak-ro, Buk-gu, Daegu 41566, Republic of Korea; breezy750@naver.com; 3 Department of Food Science and Nutrition, Pukyong National University, 45 Yongso-ro, Nam-gu, Busan 48513, Republic of Korea; k423897@naver.com (H.K.); impark-sj@naver.com (S.P.)

**Keywords:** myricitrin, *db*/*db* mice, insulin resistance, hepatic steatosis, inflammation

## Abstract

Type 2 diabetes is characterized by insulin resistance, which contributes to dysregulated glucose and lipid metabolism and is associated with chronic inflammation. While previous studies have examined the effects of myricitrin in streptozotocin-induced diabetic models, its impact on the *db*/*db* mouse, a model that better reflects insulin resistance-associated metabolic disturbances, remains unclear. In this study, mice were divided into three groups (*db*/+, *db*/*db*, and *db*/*db* + 0.02% myricitrin) and were fed their respective diets for five weeks. Myricitrin supplementation reduced fat mass, adipocyte size, and plasma leptin levels, which were elevated in *db*/*db* mice. Although myricitrin did not affect fasting blood glucose levels, it lowered plasma insulin, hemoglobin A1c, postprandial glucose levels, and the homeostasis model assessment of insulin resistance, suggesting improvements in insulin sensitivity and glucose homeostasis. Enhanced pancreatic insulin expression, along with reduced hepatic gluconeogenic enzyme activities and mRNA expression, contributed to the improved glucose homeostasis observed in myricitrin-supplemented mice. Additionally, myricitrin reduced hepatic triglyceride levels and lipid droplet accumulation by inhibiting hepatic fatty acid synthase activity. It also decreased plasma inflammatory marker levels and their mRNA expression in adipose tissue. These findings suggest that myricitrin may be a promising therapeutic candidate for type 2 diabetes.

## 1. Introduction

In 2022, the global number of diabetes patients reached 800 million, a fourfold increase since 1990, with type 2 diabetes accounting for over 95% of all cases [1]. Type 2 diabetes is a metabolic disorder characterized by hyperglycemia, which results from increased insulin resistance due to factors such as obesity and genetic predisposition [2]. As the disease progresses, insulin deficiency may also occur [2]. The liver and adipose tissue are the main tissues that respond to insulin [3]. In the liver, insulin regulates both glucose and lipid metabolism by inhibiting glucose output through the control of glucose utilization and gluconeogenesis, while simultaneously promoting lipogenesis [3]. In adipose tissue, it enhances glucose and fatty acid uptake and regulates de novo lipogenesis and lipolysis [3,4]. Therefore, in a state of insulin resistance, dysregulated glucose and lipid metabolism in these tissues contributes to type 2 diabetes and related metabolic disorders, including dyslipidemia, metabolic dysfunction-associated steatotic liver disease (MASLD), and cardiovascular diseases [2,5,6]. MASLD is the updated term for what was previously called non-alcoholic fatty liver disease (NAFLD) [7]. Type 2 diabetes patients often experience dyslipidemia, including hypertriglyceridemia and reduced high-density lipoprotein (HDL)-cholesterol [5]. Furthermore, MASLD, which is associated with increased lipid synthesis in the liver, is frequently observed in type 2 diabetes patients [6]. Among the various factors contributing to insulin resistance, fat accumulation and inflammation in adipose tissue are thought to play a key role in the development of type 2 diabetes [8].

Myricitrin, a flavonoid glycoside, is found in various plants, including the fruit, leaves, and bark of *Myrica rubra*, as well as other species [9]. Like other O- and C-linked glycosides found in flavonoids and their derivatives, myricitrin shares similar structural characteristics that contribute to its biological activity [10,11,12]. It has gained attention for its diverse pharmacological properties, such as antioxidant, anti-inflammatory, anti-atherosclerotic, hypolipidemic, and anti-obesity effects [13,14,15,16]. Moreover, several studies have demonstrated its potential in diabetes management both in vitro and in vivo [16,17,18,19]. At a low dose (5 mg/kg body weight), myricitrin reduced fasting blood glucose, homeostasis model assessment of insulin resistance (HOMA-IR), and plasma triglyceride and cholesterol levels in D-galactose-induced aged mice [17]. In a streptozotocin (STZ) + nicotinamide-induced diabetic mouse model, myricitrin encapsulated in solid lipid nanoparticles improved hyperglycemia, insulin resistance, and pancreatic apoptosis [18]. Similarly, in high-fat diet (HFD) + STZ-treated diabetic mice, myricitrin alleviated hyperglycemia, hepatic steatosis, and inflammation [16]. More recently, Dua et al. [19] demonstrated that myricitrin not only ameliorated hyperglycemia by enhancing glucose uptake in skeletal muscle via IRS-1/PI3K/Akt/GLUT4 signaling but also mitigated renal inflammation and fibrosis in HFD + STZ-treated rats. Despite the promising anti-diabetic effects of myricitrin reported in previous studies, its precise mechanisms in glucose and lipid metabolism, as well as its effect on pancreatic insulin expression, in a well-established type 2 diabetes model like *db*/*db* mice, characterized by obesity, hyperinsulinemia, insulin resistance, and MASLD, remain unclear.

Therefore, the present study aimed to evaluate the effects of myricitrin on blood glucose, hyperinsulinemia, insulin resistance, dyslipidemia, and MASLD in *db*/*db* mice. To elucidate the underlying mechanisms, we analyzed the activities and mRNA expression of key glucose- and lipid-regulating enzymes in the liver and adipose tissues. Additionally, the effects of myricitrin on pancreatic insulin expression and systemic and adipose inflammation were evaluated. Our findings demonstrate that myricitrin improves hyperinsulinemia, insulin resistance, and MASLD. These benefits are likely mediated through the suppression of gluconeogenesis and lipogenesis in the liver, coupled with a reduction in fat mass via decreased fatty acid influx and synthesis, as well as an improvement in inflammation in adipose tissue. Furthermore, myricitrin enhanced pancreatic β-cell function and insulin production, contributing to its overall anti-diabetic efficacy.

## 2. Results

### 2.1. Effects of Myricitrin on Food Intake, Body Weight, and Fat Mass in db/db Mice

**Figure 1:**
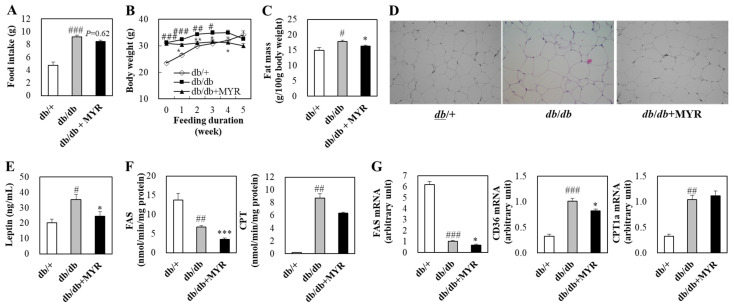
Effects of myricitrin on food intake (**A**), body weight (**B**), fat mass (**C**), histological analysis of adipose tissue (**D**), plasma leptin (**E**), lipid-regulating enzyme activity (**F**), and gene mRNA expression (**G**) in *db*/*db* mice. (**A**–**C**,**E**–**G**) Results are expressed as means ± standard error (SE) (*n* = 10 per group). # Indicates significance between *db*/+ and *db*/*db*. * Indicates significance between *db*/*db* and *db*/*db *+ MYR. # or *: *p* < 0.05, ## or **: *p* < 0.01, ### or ***: *p* < 0.001. (**D**) Representative images showing histological analysis of adipose tissue stained with hematoxylin and eosin (H&E) (Magnification, 200×). *db*/+; *db*/+ mice fed a normal diet, *db*/*db*; *db*/*db* control mice fed a normal diet, *db*/*db *+ MYR; *db*/*db* mice fed a normal diet with myricitrin (0.02%, *w*/*w*).

Food intake was significantly higher in *db*/*db* control mice than in *db*/+ mice, while the myricitrin-supplemented *db*/*db* mice showed a trend toward reduced food intake, although the difference was not statistically significant (Figure 1A). During the experimental period, *db*/+ mice exhibited continuous weight gain, whereas *db*/*db* mice gained weight until the fourth week of diet administration but showed a decline in body weight in the fifth week (Figure 1B). As a result, significant differences in body weight between *db*/+ and *db*/*db* mice were observed up to the third week. Myricitrin supplementation suppressed body weight gain in *db*/*db* mice, and their body weight was significantly lower from the first to the fourth week of supplementation compared to the *db*/*db* control group (Figure 1B).

Fat mass, which was measured by the total weight of several adipose tissues (epididymal white adipose tissue, perirenal white adipose tissue, retroperitoneal white adipose tissue, mesenteric white adipose tissue, scapular white adipose tissue, and subcutaneous white adipose tissue), was significantly higher in *db*/*db* mice than in *db*/+ mice. However, myricitrin supplementation significantly reduced fat mass compared to *db*/*db* control mice (Figure 1C). Similar to the fat mass results, hypertrophy of epididymal white adipose tissue was observed in *db*/*db* mice, whereas adipocyte size in the myricitrin-supplemented group was smaller than that of the *db*/*db* control group (Figure 1D). Additionally, plasma leptin levels were significantly higher in *db*/*db* mice than in *db*/+ mice, and myricitrin supplementation significantly reduced these levels (Figure 1E).

Although the fat mass was higher in *db*/*db* mice, the activity and mRNA expression levels of fatty acid synthase (FAS) in adipose tissue were lower in *db*/*db* mice than in *db*/+ mice, whereas the activity and mRNA expression levels of carnitine palmitoyltransferase (CPT) and the mRNA expression of CD36 were significantly higher in *db*/*db* mice than in *db*/+ mice (Figure 1F,G). In *db*/*db* mice, myricitrin supplementation significantly reduced the activities of FAS, as well as the mRNA expression of FAS and CD36 in adipose tissue (Figure 1F,G).

### 2.2. Effects of Myricitrin on Glucose Homeostasis and Insulin Regulation in db/db Mice

**Figure 2:**
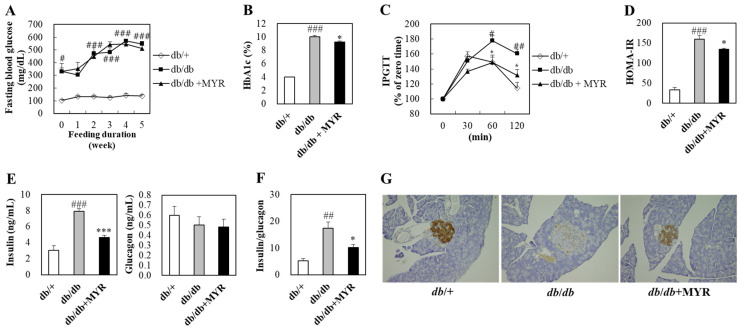
Effects of myricitrin on fasting blood glucose (**A**), HbA1c (**B**), IPGTT (**C**), HOMA-IR (**D**), plasma insulin and glucagon (**E**), insulin-to-glucagon ratio (**F**), and immunohistochemical analysis of pancreas (**G**) in *db*/*db* mice. (**A**–**F**) Results are expressed as means ± SE (*n* = 10 per group). # Indicates significance between *db*/+ and *db*/*db*. * Indicates significance between *db*/*db* and *db*/*db *+ MYR. # or *: *p* < 0.05, ##: *p* < 0.01, ### or ***: *p* < 0.001. (**D**) Representative images showing immunohistochemical staining of pancreatic islets for insulin (Magnification, 200×). *db*/+; *db*/+ mice fed a normal diet, *db*/*db*; *db*/*db* control mice fed a normal diet, *db*/*db *+ MYR; *db*/*db* mice fed a normal diet with myricitrin (0.02%, *w*/*w*).

Throughout the experimental period, fasting blood glucose levels in *db*/+ mice remained between 100 and 140 mg/dL (Figure 2A). In contrast, fasting blood glucose levels in *db*/*db* mice exceeded 300 mg/dL at baseline and continued to rise throughout the experimental period, which indicated persistent hyperglycemia (Figure 2A). Myricitrin supplementation did not significantly affect fasting blood glucose levels in *db*/*db* mice (Figure 2A). However, myricitrin significantly reduced the elevated hemoglobin A1c (HbA1c) levels in *db*/*db* mice (Figure 2B).

To assess postprandial glucose levels, an intraperitoneal glucose tolerance test (IPGTT) was conducted (Figure 2C). In *db*/+ mice, blood glucose levels peaked at 30 min after glucose injection, then declined gradually. In contrast, blood glucose levels in *db*/*db* mice peaked at 60 min, with significantly higher blood glucose levels at 60 and 120 min compared to *db*/+ mice. The myricitrin-supplemented group showed significantly lower blood glucose levels at 60 and 120 min compared to the *db*/*db* control group, indicating improved glucose regulation (Figure 2C). Consistent with the IPGTT results, HOMA-IR, an index of insulin resistance, was significantly higher in *db*/*db* mice compared to *db*/+ mice, whereas myricitrin supplementation significantly reduced HOMA-IR in *db*/*db* mice (Figure 2D).

Plasma glucagon levels were similar between *db*/+ and *db*/*db* mice, whereas plasma insulin levels were significantly higher in *db*/*db* mice, leading to a markedly increased insulin-to-glucagon ratio (Figure 2E,F). Myricitrin had no significant effect on glucagon levels but significantly reduced plasma insulin levels and the insulin-to-glucagon ratio (Figure 2E,F). Furthermore, myricitrin significantly restored pancreatic insulin expression, which was markedly reduced in *db*/*db* mice (Figure 2G).

**Figure 3:**
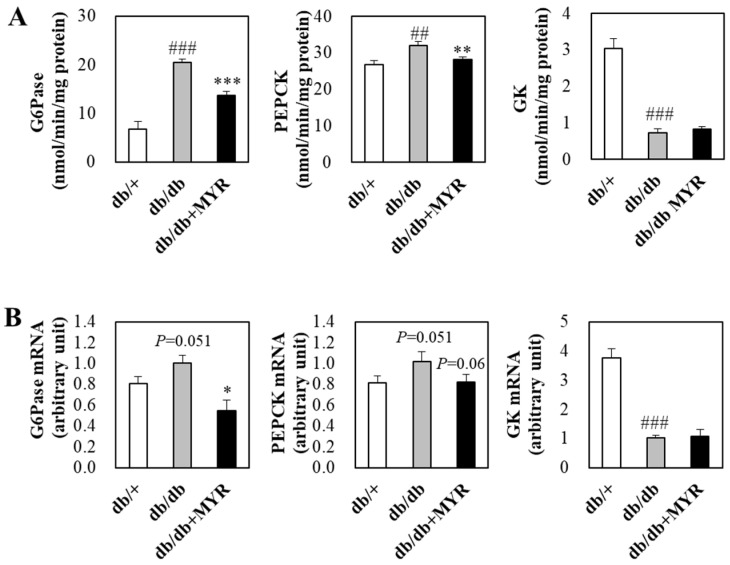
Effects of myricitrin on hepatic glucose regulating enzyme activity (**A**) and mRNA expression (**B**) in *db*/*db* mice. Results are expressed as means ± SE (*n* = 10 per group). # Indicates significance between *db*/+ and *db*/*db*. * Indicates significance between *db*/*db* and *db*/*db *+ MYR. *: *p* < 0.05, ## or **: *p* < 0.01, ### or ***: *p* < 0.001. *db*/+; *db*/+ mice fed a normal diet, *db*/*db*; *db*/*db* control mice fed a normal diet, *db*/*db *+ MYR; *db*/*db* mice fed a normal diet with myricitrin (0.02%, *w*/*w*).

In *db*/*db* mice, the activities of gluconeogenic enzymes, glucose-6-phosphatase (G6Pase) and phosphoenolpyruvate carboxykinase (PEPCK), were significantly higher, while the activity of the glycolytic enzyme glucokinase (GK) was significantly lower compared to *db*/+ mice (Figure 3A). Myricitrin supplementation significantly inhibited the elevated hepatic G6Pase and PEPCK activities in *db*/*db* mice but had no significant effect on hepatic GK activity (Figure 3A). Consistent with these changes in hepatic glucose-regulating enzyme activities, myricitrin supplementation down-regulated the increased mRNA expression of G6Pase and PEPCK in *db*/*db* mice but did not affect GK mRNA expression (Figure 3B).

### 2.3. Effects of Myricitrin on Lipid Homeostasis and Liver Morphology in db/db Mice

**Figure 4:**
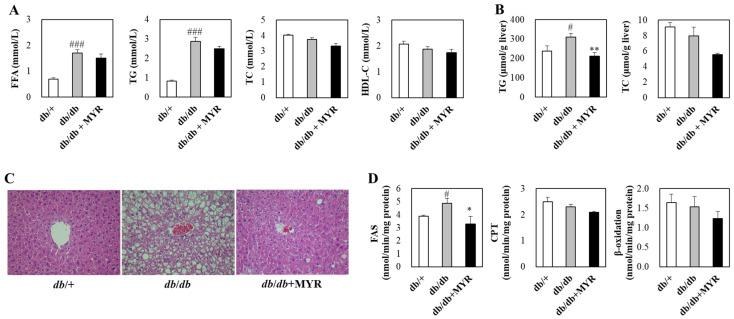
Effects of myricitrin on plasma (**A**) and hepatic lipids levels (**B**), hepatic histological analysis (**C**), and hepatic lipid regulating enzyme activity (**D**) in *db*/*db* mice. (**A**,**B**,**D**) Results are expressed as means ± SE (*n* = 10 per group). # Indicates significance between *db*/+ and *db*/*db*. * Indicates significance between *db*/*db* and *db*/*db *+ MYR. # or *: *p* < 0.05, **: *p* < 0.01, ###: *p* < 0.001. (**C**) Representative images showing histological analysis of liver stained with H&E (Magnification, 200×). *db*/+; *db*/+ mice fed a normal diet, *db*/*db*; *db*/*db* control mice fed a normal diet, *db*/*db *+ MYR; *db*/*db* mice fed a normal diet with myricitrin (0.02%, *w*/*w*), FFA; free fatty acid, TG; triglyceride, TC; total cholesterol, HDL-C; HDL-cholesterol.

Plasma-free fatty acid and triglyceride concentrations were significantly higher in *db*/*db* mice compared to *db*/+ mice, while total cholesterol and HDL-cholesterol levels did not differ between the two groups (Figure 4A). Myricitrin supplementation did not induce significant changes in these plasma lipids concentrations in *db*/*db* mice (Figure 4A). However, myricitrin significantly reduced hepatic triglyceride levels, which were markedly elevated in *db*/*db* mice compared to *db*/+ mice (Figure 4B). Hepatic cholesterol levels were also reduced by approximately 30% in myricitrin-supplemented *db*/*db* mice compared to the *db*/*db *control group, although this difference did not reach statistical significance (Figure 4B). Histological analysis revealed that the livers of *db*/*db* control mice exhibited numerous lipid droplets of varying sizes surrounding the portal vein, whereas myricitrin supplementation significantly reduced the accumulation of hepatic lipid droplets, restoring them to levels comparable to those observed in *db*/+ mice (Figure 4C).

The activity of FAS, an enzyme involved in fatty acid synthesis, was significantly higher in *db*/*db* mice compared to *db*/+ mice, while the activities of CPT and β-oxidation, which are involved in fatty acid oxidation, did not differ between the two groups (Figure 4D). Myricitrin supplementation in *db*/*db* mice did not significantly affect the activities of hepatic CPT and β-oxidation but significantly reduced FAS activity in the liver (Figure 4D).

### 2.4. Effects of Myricitrin on Circulating and Adipose Inflammatory Markers in db/db Mice

**Figure 5:**
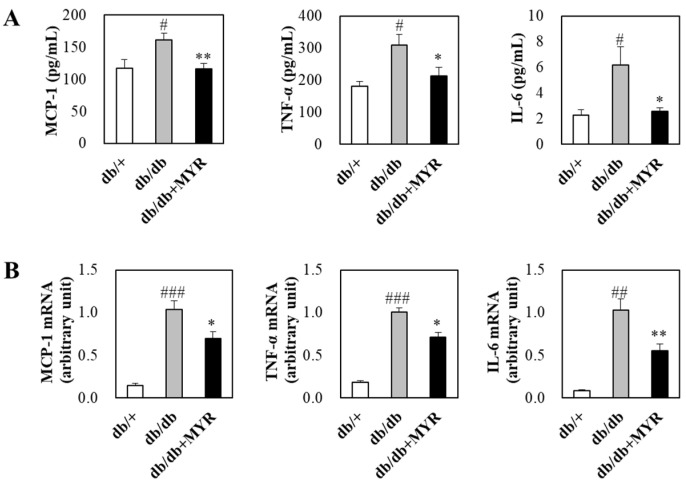
Effects of myricitrin on plasma levels of pro-inflammatory markers (**A**) and their mRNA expression in adipose tissue (**B**) of *db*/*db* mice. Results are expressed as means ± SE (*n* = 10 per group). # Indicates significance between *db*/+ and *db*/*db*. * Indicates significance between *db*/*db* and *db*/*db *+ MYR. # or *: *p* < 0.05, ## or **: *p* < 0.01, ###: *p* < 0.001. *db*/+; *db*/+ mice fed a normal diet, *db*/*db*; *db*/*db* control mice fed a normal diet, *db*/*db *+ MYR; *db*/*db* mice fed a normal diet with myricitrin (0.02%, *w*/*w*).

Plasma concentrations of inflammatory markers, including monocyte chemoattractant protein (MCP)-1, tumor necrosis factor (TNF)-α, and interleukin (IL)-6, were significantly higher in *db*/*db* mice compared to *db*/+ mice (Figure 5A). However, myricitrin supplementation significantly reduced the levels of these inflammatory markers in plasma (Figure 5A). Additionally, the mRNA expression levels of MCP-1, TNF-α, and IL-6 in adipose tissue were significantly up-regulated in *db*/*db* mice compared to *db*/+ mice. Myricitrin supplementation effectively suppressed the increased expression of these inflammatory genes in *db*/*db* mice (Figure 5A).

## 3. Discussion

The *db*/*db* mouse model is particularly valuable in type 2 diabetes research due to its natural progression of key characteristics, including obesity, hyperglycemia, insulin resistance, and MASLD [20,21]. While myricitrin has been reported to exert anti-diabetic effects in various animal models induced by substances such as STZ or D-galactose, which trigger acute forms of diabetes or aging, its impact and underlying mechanisms in *db*/*db* mice remain unexplored. Therefore, the present study aimed to investigate the effects of myricitrin supplementation on key metabolic disturbances in *db*/*db* mice, focusing specifically on insulin resistance, hepatic steatosis, and inflammation.

This study demonstrates that myricitrin supplementation significantly improves insulin sensitivity and glucose homeostasis in *db*/*db* mice. The reduction in HOMA-IR and improved glucose tolerance in IPGTT indicate that myricitrin effectively alleviates insulin resistance and improves glucose homeostasis [22,23]. Moreover, myricitrin supplementation significantly reduced HbA1c levels, although fasting blood glucose levels remained unchanged. Since HbA1c reflects average blood glucose levels over the past 2–3 months [24], these findings suggest that myricitrin exerts sustained beneficial effects on glucose homeostasis despite the lack of immediate fasting glucose reduction. HbA1c is also recognized as a simple and useful marker for accurately predicting insulin sensitivity in diabetes patients [24]. The lack of fasting glucose reduction could be attributed to the severe insulin resistance in *db*/*db* mice, which limits the glucose-lowering effects of interventions. Similar findings have been reported in previous studies conducted in models of insulin resistance, such as type 2 diabetes and obesity [18,25,26,27].

The improvement in insulin resistance observed in this study is closely linked to reductions in hepatic glucose production and lipid accumulation. Under normal physiological conditions, insulin suppresses hepatic gluconeogenesis. However, in type 2 diabetes, insulin resistance disrupts this process, leading to sustained glucose production in the liver [28]. Hepatic glucose production is the primary source of endogenous gluconeogenesis, contributing approximately 90% [29], underscoring its essential role in glucose homeostasis [30]. The inability of insulin to effectively inhibit hepatic glucose production has been identified as a key factor in the development of type 2 diabetes [31]. Gluconeogenesis in the liver is primarily regulated by two key enzymes, PEPCK and G6Pase [32]. G6Pase catalyzes the final step in gluconeogenesis, while PEPCK facilitates the conversion of oxaloacetate to phosphoenolpyruvate, a critical rate-limiting step. Studies have shown that the expression and activity of these enzymes are elevated in the liver of type 2 diabetic individuals and animal models such as *db*/*db* mice [33,34,35,36]. In this study, the activities of PEPCK and G6Pase were significantly higher in *db*/*db* mice than in *db*/+ mice. However, supplementation with myricitrin significantly reduced the activities of both enzymes. Consistent with these findings, myricitrin also lowered the mRNA expression of these gluconeogenic enzymes.

Additionally, myricitrin supplementation improved hepatic steatosis, as evidenced by decreased hepatic triglyceride accumulation and lipid droplet formation. Type 2 diabetes patients often develop MASLD [6], which is associated with an increased risk of insulin resistance [37]. In type 2 diabetes, impaired suppression of hepatic gluconeogenesis is linked to excessive de novo lipogenesis, leading to lipid accumulation and MASLD development [36,38]. Although insulin promotes fatty acid synthesis, de novo lipogenesis in the liver is further enhanced in insulin resistance, contributing to MASLD [37]. FAS, which catalyzes the transformation of acetyl-CoA and malonyl-CoA into palmitate, plays a crucial role in de novo lipogenesis [39]. Therefore, the inhibition of FAS activity by myricitrin may contribute to reduced de novo lipogenesis, ultimately alleviating hepatic steatosis, and is likely related to the improvement of insulin resistance.

Furthermore, our results demonstrate that myricitrin enhances pancreatic insulin expression while simultaneously lowering circulating insulin levels. Unlike type 1 diabetes, which is characterized by insulin deficiency, type 2 diabetes initially leads to increased insulin secretion as a compensatory response to elevated blood glucose levels, resulting in hyperinsulinemia [2]. However, as insulin resistance persists, pancreatic β-cell dysfunction and loss occur, ultimately leading to inadequate insulin production and worsening hyperglycemia [40]. In *db*/*db* mice, plasma insulin levels increase from 4 to 12 weeks of age but decline at 16 weeks [41]. Nevertheless, insulin levels remain higher than those in *db*/+ mice, indicating persistent hyperinsulinemia [41]. This persistent hyperinsulinemia, however, is accompanied by reduced pancreatic insulin expression due to pancreatic β-cell dysfunction [42]. Consistent with these findings, our study showed that plasma insulin levels were significantly higher in *db*/*db* mice than in *db*/+ mice, indicating persistent hyperinsulinemia. However, immunohistochemistry analysis revealed lower pancreatic insulin expression in *db*/*db* mice. These results imply that despite compensatory hyperinsulinemia in response to insulin resistance, pancreatic β-cell function is gradually deteriorating. Notably, myricitrin may help delay β-cell exhaustion and mitigate hyperinsulinemia by improving insulin resistance. Although the present study did not directly investigate the mechanisms by which myricitrin enhances pancreatic insulin expression, its antioxidant and anti-inflammatory properties may contribute to β-cell protection and improved insulin secretion [43,44,45].

Obesity is a major risk factor for insulin resistance and type 2 diabetes. In obesity, increased adipose tissue and inflammatory responses in adipose tissue are linked to insulin resistance and hyperinsulinemia [46,47]. In the present study, myricitrin decreased CD36 mRNA expression, along with FAS activity and mRNA expression in adipose tissue. CD36 is a key transporter involved in fatty acid uptake [48]. The inhibition of CD36 or its receptor has been reported to protect against obesity, insulin resistance, and inflammation [49,50]. Therefore, the decreased fat mass and improved adipocyte hypertrophy observed in myricitrin-supplemented mice may be attributed to reductions in fatty acid synthesis and uptake in adipose tissue, contributing to improved insulin sensitivity.

Moreover, myricitrin supplementation significantly reduced plasma MCP-1, TNF-α, and IL-6 levels, as well as their mRNA expression in adipose tissue, which was markedly elevated in *db*/*db* mice compared to *db*/+ mice. Chronic inflammation in adipose tissue is a key contributor to insulin resistance in type 2 diabetes, along with excessive fat accumulation [8,47]. Pro-inflammatory chemokines and cytokines, such as MCP-1, TNF-α, and IL-6, can induce insulin resistance through various mechanisms, including inflammation promotion and impairment of insulin sensitivity [8]. TNF-α is a well-known cytokine that induces insulin resistance [51]. TNF-α expression was increased in the adipose tissue of type 2 diabetic individuals and *db*/*db* mice [52,53], and the loss of TNF-α function protected against obesity-induced insulin resistance [54]. In *db*/*db* mice, mRNA expression and plasma levels of other pro-inflammatory markers, such as MCP-1 and IL-6, were also elevated [55,56]. MCP-1 overexpression in adipose tissue exacerbated insulin resistance, whereas its inhibition alleviated HFD-induced insulin resistance and hepatic steatosis [55]. Moreover, adipose tissue IL-6 levels showed an inverse correlation with insulin sensitivity in obese individuals with type 2 diabetes [57], and IL-6 released from adipose tissue contributed to hepatic insulin resistance [58,59]. Since adipose tissue inflammation and insulin resistance are closely interconnected, the improvement in insulin resistance observed in myricitrin-supplemented mice is likely associated with reduced adipose tissue inflammation and fat mass.

Another notable finding is that while the body weight of *db*/+ mice steadily increased throughout the study, *db*/*db* mice typically exhibited progressive weight gain until around the fourth week of experimental interventions (9 weeks of age), after which body weight began to decline. Similarly, Orland and Permutt [60] reported that *db*/*db* mice start losing weight around 10 weeks of age, coinciding with diabetes progression. Furthermore, food intake in *db*/*db* mice was significantly higher than in *db*/+ mice, indicating polyphagia, a common characteristic of diabetes. In contrast, myricitrin supplementation for 5 weeks slightly reduced food intake and effectively prevented excessive weight gain throughout the study. This regulation of body weight, possibly through the modulation of lipid metabolism and reduction of adiposity, likely contributed to the observed improvement in insulin sensitivity. Additionally, myricitrin supplementation reduced plasma leptin levels, which were elevated in *db*/*db* mice. Since hyperleptinemia in obesity is associated with impaired body weight regulation, energy homeostasis, and glucose intolerance [61], the decrease in leptin levels may further support the metabolic benefits of myricitrin.

Given the rapid disease progression in *db*/*db* mice, the current study focused on evaluating early-stage metabolic improvements. However, a longer period of study would provide further insights into the long-term effects and potential safety of myricitrin supplementation. Another limitation of this study is the absence of a dose–response analysis, which would be important to determine the optimal dosage of myricitrin for maximizing metabolic improvements and provide a more comprehensive understanding of its efficacy. Additionally, although the *db*/*db* model is widely used for studying insulin resistance-associated metabolic disturbances, interspecies differences between the mouse model and human subjects with type 2 diabetes should be considered. In mice, myricitrin is absorbed in the gastrointestinal tract and hydrolyzed by intestinal β-glucosidases, releasing the aglycone myricetin [62]. Myricetin is then absorbed into the bloodstream, metabolized in the liver into water-soluble sulfates and glucuronides, and excreted through urine and bile. Although similar metabolic processes occur in humans, differences in enzyme activity, gut microbiota, and absorption mechanisms may affect myricitrin bioavailability and pharmacokinetics in humans [62,63]. Moreover, a low bioavailability of myricitrin, due to poor water solubility, gastrointestinal tract instability, and conversion to myricetin by colonic microflora, may limit its efficacy [64,65,66]. Future studies should adopt complementary models to enhance translational relevance, optimize bioavailability, and compare efficacy with standard anti-diabetic drugs to better assess myricitrin’s clinical potential.

## 4. Materials and Methods

### 4.1. Animal and Diet

Male C57BL/KsJ-*db*/*db* mice (4 weeks old) were purchased from Jackson Laboratory (Bar Harbor, ME, USA). Each mouse was housed individually in an environmentally controlled animal facility with automatic settings maintaining a temperature of 25 ± 2 °C, a humidity of 50 ± 5%, and a 12-h light-dark cycle (08:00–20:00). During the one-week acclimatization period, the animals were fed a chow diet. After acclimatization, the animals were assigned to three groups (*n* = 10 per group) using a randomized block design, ensuring similar baseline blood glucose levels and body weight among groups. The groups were as follows: the normal group (C57BL/KsJ-*db*/+ mice, *db*/+) fed an AIN-76 semisynthetic diet [67,68], the diabetic control group (C57BL/KsJ-*db*/*db* mice, *db*/*db*) fed the same normal diet, and the experimental group (*db*/*db* + MYR) fed the normal diet supplemented with 0.02% (*w*/*w*) myricitrin for five weeks. The experimental diets in powder form and drinking water were provided ad libitum. Food intake was recorded daily by measuring leftover food, and body weight and blood glucose levels were measured weekly. The animal experiment was conducted with the approval of the Institutional Animal Care and Use Committee of Pukyong National University (2017-45).

### 4.2. Blood Collection and Organ Harvesting

At the end of the experimental period, the animals were fasted for 12 h before sacrifice. They were anesthetized with isoflurane (5 mg/kg body weight, Baxter, Deerfield, IL, USA), and blood was collected from the inferior vena cava. A portion of the whole blood was used for fasting blood glucose and HbA1c analyses, while the remaining blood was treated with heparin and centrifuged at 1000× *g* for 15 min at 4 °C to obtain plasma. The pancreas, liver, and various adipose tissues (epididymal, perirenal, retroperitoneal, mesenteric, scapular, and subcutaneous white adipose tissues) were immediately excised, rinsed with physiological saline, and blotted to remove excess moisture. Liver and adipose tissues were rapidly frozen in liquid nitrogen and stored at −70 °C for further analysis.

### 4.3. Fasting Blood Glucose, HbA1c, Plasma Insulin, Glucagon, Leptin, and Inflammatory Markers

Fasting blood glucose levels were measured using a glucometer (Gluco Dr SuperSensor, Allmedicus, Anyang, Republic of Korea) with blood samples obtained from the tail vein after a 12-h fast. HbA1c levels were measured using a glycated hemoglobin analyzer (Micromat™ I Hemoglobin A1c Test, Bio-Rad, Hercules, CA, USA) with blood collected from the inferior vena cava after a 12-h fast. Plasma insulin, glucagon, leptin, MCP-1, TNF-α, and IL-6 levels were quantified using a multiplex detection kit (Bio-Rad) on a Luminex 200 Labmap system (Bio-Rad). HOMA-IR was calculated using the following formula:

HOMA-IR = [fasting glucose (mmol/L) × fasting insulin (µU/mL)]/2.51

### 4.4. Intraperitoneal Glucose Tolerance Test

To evaluate glucose tolerance, an IPGTT was conducted in the fourth week of dietary intervention. After a 12-h fast, a glucose solution was administered intraperitoneally at a dose of 0.5 g/kg body weight. Blood glucose levels were measured at 0 (fasting glucose), 30, 60, and 120 min post-glucose administration using a glucometer (Gluco Dr SuperSensor, All Medicus Co., Ltd., Seoul, Republic of Korea) with blood samples collected from the tail vein.

### 4.5. Plasma and Hepatic Lipid Levels

Plasma-free fatty acid was quantified using a non-esterified fatty acid assay kit (Wako Chemicals, Richmond, VA, USA). Plasma triglyceride, total cholesterol, and HDL-cholesterol levels were determined using specific assay kits (Asan Pharm. Co., Ltd., Seoul, Republic of Korea).

Hepatic lipids were extracted using the Folch method [69]. Briefly, 0.5 g of liver tissue was finely minced and homogenized in 5 mL of a chloroform/methanol (2:1) solution. The lipid extract was filtered, dried under nitrogen gas, and redissolved in 1 mL of the extraction solvent. Aliquots were taken for triglyceride and total cholesterol analyses using the same enzymatic methods applied to plasma lipid measurements.

### 4.6. Enzyme Activity in Liver and Adipose Tissues

The activities of enzymes involved in glucose and lipid metabolism in liver and adipose tissue were measured using methods adapted from Kim et al. [70]. To measure glucose metabolism enzyme activity in the liver, GK activity was assessed by monitoring NAD⁺ reduction to NADH, based on a modified method of Davidson and Arion [71]. The reaction mixture contained HEPES (pH 7.4), KCl, dithioerythritol, MgCl_2_, BSA, NAD⁺, glucose-6-phosphate dehydrogenase, glucose, and ATP. Cytosolic enzyme fraction was added, and absorbance was measured at 340 nm for 10 min at 37 °C. GK activity was expressed as nmol NADH produced per minute per mg of protein. G6Pase activity was determined using a modified method of Alegre et al. [72]. The mixture containing HEPES (pH 6.5), glucose-6-phosphate, EDTA, NADP⁺, mutarotase, and glucose dehydrogenase was incubated for 4 min at 37 °C, after which the microsomal fraction was added, and the reaction continued for another 4 min. Absorbance was measured at 340 nm. G6Pase activity was expressed as nmol NADPH produced per minute per mg of microsomal protein. PEPCK activity was measured by monitoring oxaloacetate production using the Bentle and Lardy [73] method. The reaction mixture contained HEPES (pH 6.5), inosine-5′-diphosphate trisodium, MnCl_2_, dithiothreitol, NADH, phosphoenolpyruvate, NaHCO_3_, and L-malate dehydrogenase. Cytosolic enzyme extract was added, and absorbance was measured at 340 nm for 2 min at 25 °C. PEPCK activity was expressed as nmol NAD⁺ produced per minute per mg of protein.

For the measurement of lipid metabolism enzyme activity in the liver and adipose tissue, FAS activity was measured using a modified method of Nepokroeff et al. [74]. The reaction mixture consisted of potassium phosphate buffer (pH 7.0), EDTA, β-mercaptoethanol, acetyl-CoA, malonyl-CoA, NADPH, and cytosolic fraction. The reaction was carried out at 30 °C for 10 min, and the decrease in absorbance was measured at 340 nm. FAS activity was expressed as nmol NADPH oxidized per minute per mg of protein. CPT activity was assessed using the method of Markwell et al. [75], which measures CoASH production from palmitoyl-CoA. The reaction mixture included Tris-HCl (pH 8.0), EDTA, L-carnitine, 5,5′-Dithiobis(2-nitrobenzoic acid), palmitoyl-CoA, and Triton X-100. The mitochondrial fraction was added, and absorbance was measured at 412 nm for 2 min at 25 °C. β-oxidation activity was determined with NADH production from palmitoyl-CoA, using the method of Lazarow [76]. The reaction mixture consisted of Tris-HCl (pH 8.0), NAD⁺, dithiothreitol, BSA, Triton X-100, Coenzyme A, flavin adenine dinucleotide, potassium cyanide, and palmitoyl-CoA. The mitochondrial fraction was added, and absorbance was measured at 340 nm for 5 min at 37 °C. β-oxidation activity was expressed as nmol NADH produced per minute per mg of mitochondrial protein. Protein concentrations for enzyme activity normalization were determined using the Bradford method [77].

### 4.7. RNA Extraction and Real-Time qPCR

Total RNA was extracted using TRIzol reagent (Invitrogen Life Technologies, Grand Island, NY, USA). The concentration and purity of the isolated RNA were determined by measuring absorbance at 260 nm and 280 nm with a NanoDrop 1000 spectrophotometer (Thermo Fisher Scientific, Waltham, MA, USA). mRNA expression levels were analyzed with real-time quantitative polymerase chain reaction (qPCR) using a SYBR Green PCR kit (Qiagen, Hilden, Germany). Real-time qPCR was performed on a CFX96™ real-time PCR system (Bio-Rad), and threshold cycle (Ct) values were determined using CFX3.1 software (Bio-Rad). The expression levels of target genes were normalized to GAPDH expression from the same sample, and relative mRNA expression was calculated using the 2^−ΔΔCt^ method [78].

### 4.8. Histopathological Analysis of Pancreatic, Liver, and Adipose Tissues

Pancreatic tissue fixed in 10% formaldehyde solution was subjected to immunohistochemical staining to assess insulin expression using a primary anti-insulin antibody (Santa Cruz Biotech, Inc., Dallas, TX, USA). Antibody reactivity was detected using a horseradish peroxidase-conjugated biotin–streptavidin complex and visualized with diaminobenzidine as the chromogenic substrate. Liver and epididymal white adipose tissues were stained with H&E to examine histological changes under a light microscope at 200× magnification.

### 4.9. Statistical Analysis

All data were statistically analyzed using SPSS, version 22 (Statistical Package for the Social Sciences) software. Data were presented as mean ± SE. Statistical significance was considered at *p *< 0.05. Differences in mean values between the normal (*db*/+) group and diabetic control (*db*/*db*) group, as well as between the diabetic control (*db*/*db*) group and experimental (*db*/*db *+ MYR) group, were analyzed using Student’s *t*-test.

## 5. Conclusions

Our study provides compelling evidence that myricitrin exerts multifaceted metabolic benefits by improving insulin resistance and hyperinsulinemia, reducing hepatic glucose production and lipid accumulation, and preserving pancreatic function. Additionally, myricitrin reduced fat mass and inflammation in adipose tissue, potentially enhancing insulin sensitivity. These findings suggest that myricitrin may have therapeutic potential for managing metabolic disorders related to type 2 diabetes. Further research is needed to elucidate the precise molecular mechanisms involved, assess long-term efficacy, and evaluate its potential application in clinical settings.

## Data Availability

The original contributions presented in this study are included in the article. Further inquiries can be directed to the corresponding author.

## Abbreviations

The following abbreviations are used in this manuscript:

MASLD	Metabolic dysfunction-associated steatotic liver disease
HDL	High-density lipoprotein
HOMA-IR	Homeostasis model assessment of insulin resistance
STZ	Streptozotocin
HFD	High-fat diet
FAS	Fatty acid synthase
CPT	Carnitine palmitoyltransferase
HbA1c	Hemoglobin A1c
IPGTT	Intraperitoneal glucose tolerance test
G6Pase	Glucose-6-phosphatase
PEPCK	Phosphoenolpyruvate carboxykinase
GK	Glucokinase
MCP-1	Monocyte chemoattractant protein-1
TNF-α	Tumor necrosis factor-α
IL-6	Interleukin-6

## References

[1] NCD Risk Factor Collaboration (NCD-RisC) (2024). Worldwide trends in diabetes prevalence and treatment from 1990 to 2022: A pooled analysis of 1108 population-representative studies with 141 million participants. Lancet.

[2] Galicia-Garcia U., Benito-Vicente A., Jebari S., Larrea-Sebal A., Siddiqi H., Uribe K.B., Ostolaza H., Martín C. (2020). Pathophysiology of Type 2 Diabetes Mellitus. Int. J. Mol. Sci..

[3] Fazakerley D.J., Krycer J.R., Kearney A.L., Hocking S.L., James D.E. (2019). Muscle and adipose tissue insulin resistance: Malady without mechanism?. J. Lipid Res..

[4] Santoro A., McGraw T.E., Kahn B.B. (2021). Insulin action in adipocytes, adipose remodeling, and systemic effects. Cell Metab..

[5] Chait A., Eckel R.H., Vrablik M., Zambon A. (2024). Lipid-lowering in diabetes: An update. Atherosclerosis.

[6] Nogueira J.P., Cusi K. (2024). Role of insulin resistance in the development of nonalcoholic fatty liver disease in people with type 2 diabetes: From bench to patient care. Diabetes Spectr..

[7] Bae J.C. (2024). No more NAFLD: The term is now MASLD. Endocrinol. Metab..

[8] Burhans M.S., Hagman D.K., Kuzma J.N., Schmidt K.A., Kratz M. (2018). Contribution of adipose tissue inflammation to the development of type 2 diabetes mellitus. Compr. Physiol..

[9] Huang Q., Gao B., Wang L., Hu Y.Q., Lu W.G., Yang L., Luo Z.J., Liu J. (2014). Protective effects of myricitrin against osteoporosis via reducing reactive oxygen species and bone-resorbing cytokines. Toxicol. Appl. Pharmacol..

[10] Sudarshan K., Aidhen I.S. (2017). Convenient synthesis of 3-glycosylated isocoumarins. Eur. J. Org. Chem..

[11] Hasegawa T., Tanaka A., Hosoda A., Takano F., Ohta T. (2008). Antioxidant C-glycosyl flavones from the leaves of Sasa kurilensis var. gigantea. Phytochemistry.

[12] Sato S., Hiroe K., Kumazawa T., Jun-ichi O. (2006). Total synthesis of two isoflavone C-glycosides: Genistein and orobol 8-C-beta-D-glucopyranosides. Carbohydr. Res..

[13] Wang M., Sun G.B., Du Y.Y., Tian Y., Liao P., Liu X.S., Ye J.X., Sun X.B. (2017). Myricitrin protects cardiomyocytes from hypoxia/reoxygenation injury: Involvement of heat shock protein 90. Front. Pharmacol..

[14] Domitrovic R., Rashed K., Cvijanovic O., Vladimir-Knezevic S., Skoda M., Visnic A. (2015). Myricitrin exhibits antioxidant, anti-inflammatory and antifibrotic activity in carbon tetrachloride-intoxicated mice. Chem. Biol. Interact..

[15] Gao J., Liu C., Zhang H., Sun Z., Wang R. (2019). Myricitrin exhibits anti-atherosclerotic and anti-hyperlipidemic effects in diet-induced hypercholesterolemic rats. AMB Express.

[16] Kim Y.J., Kim S.R., Kim D.Y., Woo J.T., Kwon E.Y., Han Y., Choi M.S., Jung U.J. (2019). Supplementation of the flavonoid myricitrin attenuates the adverse metabolic effects of long-term consumption of a high-fat diet in mice. J. Med. Food.

[17] Omidi M., Ahangarpour A., Khorsandi L., Ramezani-AliAkbari F. (2020). The antidiabetic and hepatoprotective effects of myricitrin on aged mice with D-galactose. Gastroenterol. Hepatol. Bed Bench.

[18] Ahangarpour A., Oroojan A.A., Khorsandi L., Kouchak M., Badavi M. (2018). Solid lipid nanoparticles of myricitrin have antioxidant and antidiabetic effects on streptozotocin-nicotinamide-induced diabetic model and myotube cell of male mouse. Oxid. Med. Cell Longev..

[19] Dua T.K., Joardar S., Chakraborty P., Bhowmick S., Saha A., De Feo V., Dewanjee S. (2021). Myricitrin, a glycosyloxyflavone in Myrica esculenta bark ameliorates diabetic nephropathy via improving glycemic status, reducing oxidative stress, and suppressing inflammation. Molecules.

[20] Belke D.D., Severson D.L. (2012). Diabetes in mice with monogenic obesity: The *db*/*db* mouse and its use in the study of cardiac consequences. Methods Mol. Biol..

[21] Kim K.E., Jung Y., Min S., Nam M., Heo R.W., Jeon B.T., Song D.H., Yi C.O., Jeong E.A., Kim H. (2016). Caloric restriction of *db*/*db* mice reverts hepatic steatosis and body weight with divergent hepatic metabolism. Sci. Rep..

[22] Bonora E., Formentini G., Calcaterra F., Lombardi S., Marini F., Zenari L., Saggiani F., Poli M., Perbellini S., Raffaelli A. (2002). HOMA-estimated insulin resistance is an independent predictor of cardiovascular disease in type 2 diabetic subjects: Prospective data from the Verona Diabetes Complications Study. Diabetes Care.

[23] Al Rijjal D., Wheeler M.B. (2022). A protocol for studying glucose homeostasis and islet function in mice. STAR Protoc..

[24] Sherwani S.I., Khan H.A., Ekhzaimy A., Masood A., Sakharkar M.K. (2016). Significance of HbA1c test in diagnosis and prognosis of diabetic patients. Biomark. Insights.

[25] Esterson Y.B., Zhang K., Koppaka S., Kehlenbrink S., Kishore P., Raghavan P., Maginley S.R., Carey M., Hawkins M. (2013). Insulin sensitizing and anti-inflammatory effects of thiazolidinediones are heightened in obese patients. J. Investig. Med..

[26] Schenk S., Harber M.P., Shrivastava C.R., Burant C.F., Horowitz J.F. (2009). Improved insulin sensitivity after weight loss and exercise training is mediated by a reduction in plasma fatty acid mobilization, not enhanced oxidative capacity. J. Physiol..

[27] Sutton E.F., Beyl R., Early K.S., Cefalu W.T., Ravussin E., Peterson C.M. (2018). Early time-restricted feeding improves insulin sensitivity, blood pressure, and oxidative stress even without weight loss in men with prediabetes. Cell Metab..

[28] Bo T., Gao L., Yao Z., Shao S., Wang X., Proud C.G., Zhao J. (2024). Hepatic selective insulin resistance at the intersection of insulin signaling and metabolic dysfunction-associated steatotic liver disease. Cell Metab..

[29] Ekberg K., Landau B.R., Wajngot A., Chandramouli V., Efendic S., Brunengraber H., Wahren J. (1999). Contributions by kidney and liver to glucose production in the postabsorptive state and after 60 h of fasting. Diabetes.

[30] Sharabi K., Tavares C.D., Rines A.K., Puigserver P. (2015). Molecular pathophysiology of hepatic glucose production. Mol. Aspects Med..

[31] Cherrington A.D. (2005). The role of hepatic insulin receptors in the regulation of glucose production. J. Clin. Investig..

[32] Yabaluri N., Bashyam M.D. (2010). Hormonal regulation of gluconeogenic gene transcription in the liver. J. Biosci..

[33] Altomonte J., Richter A., Harbaran S., Suriawinata J., Nakae J., Thung S.N., Meseck M., Accili D., Dong H. (2003). Inhibition of Foxo1 function is associated with improved fasting glycemia in diabetic mice. Am. J. Physiol. Endocrinol. Metab..

[34] Cao H., Veer E., Ban M.R., Hanley A.J., Zinman B., Harris S.B., Young T.K., Pickering J.G., Hegele R.A. (2004). Promoter polymorphism in PCK1 (phosphoenolpyruvate carboxykinase gene) associated with type 2 diabetes mellitus. J. Clin. Endocrinol. Metab..

[35] Liu Y., Nakagawa Y., Wang Y., Sakurai R., Tripathi P.V., Lutfy K., Friedman T.C. (2005). Increased glucocorticoid receptor and 11{beta}-hydroxysteroid dehydrogenase type 1 expression in hepatocytes may contribute to the phenotype of type 2 diabetes in *db*/*db* mice. Diabetes.

[36] Gómez-Valadés A.G., Méndez-Lucas A., Vidal-Alabró A., Blasco F.X., Chillon M., Bartrons R., Bermúdez J., Perales J.C. (2008). Pck1 gene silencing in the liver improves glycemia control, insulin sensitivity, and dyslipidemia in *db*/*db* mice. Diabetes.

[37] Smith G.I., Shankaran M., Yoshino M., Schweitzer G.G., Chondronikola M., Beals J.W., Okunade A.L., Patterson B.W., Nyangau E., Field T. (2020). Insulin resistance drives hepatic de novo lipogenesis in nonalcoholic fatty liver disease. J. Clin. Investig..

[38] Onyango A.N. (2022). Excessive gluconeogenesis causes the hepatic insulin resistance paradox and its sequelae. Heliyon.

[39] Berndt J., Kovacs P., Ruschke K., Klöting N., Fasshauer M., Schön M.R., Körner A., Stumvoll M., Blüher M. (2007). Fatty acid synthase gene expression in human adipose tissue: Association with obesity and type 2 diabetes. Diabetologia.

[40] Talchai C., Xuan S., Lin H.V., Sussel L., Accili D. (2012). Pancreatic β cell dedifferentiation as a mechanism of diabetic β cell failure. Cell.

[41] Okajima Y., Matsuzaka T., Miyazaki S., Motomura K., Ohno H., Sharma R., Shimura T., Istiqamah N., Han S.I., Mizunoe Y. (2022). Morphological and functional adaptation of pancreatic islet blood vessels to insulin resistance is impaired in diabetic *db*/*db* mice. Biochim. Biophys. Acta-Mol. Basis Dis..

[42] Matsuoka T.A., Kaneto H., Miyatsuka T., Yamamoto T., Yamamoto K., Kato K., Shimomura I., Stein R., Matsuhisa M. (2010). Regulation of MafA expression in pancreatic beta-cells in *db*/*db* mice with diabetes. Diabetes.

[43] Karunakaran U., Park K.-G. (2013). A systematic review of oxidative stress and safety of antioxidants in diabetes: Focus on islets and their defense. Diabetes Metab. J..

[44] Kuryłowicz A., Koźniewski K. (2020). Anti-inflammatory strategies targeting metaflammation in type 2 diabetes. Molecules.

[45] Ghorbani A., Rashidi R., Shafiee-Nick R. (2019). Flavonoids for preserving pancreatic beta cell survival and function: A mechanistic review. Biomed. Pharmacother..

[46] Zatterale F., Longo M., Naderi J., Raciti G.A., Desiderio A., Miele C., Beguinot F. (2020). Chronic adipose tissue inflammation linking obesity to insulin resistance and type 2 diabetes. Front. Physiol..

[47] Kawai T., Autieri M.V., Scalia R. (2021). Adipose tissue inflammation and metabolic dysfunction in obesity. Am. J. Physiol. Cell Physiol..

[48] Coburn C.T., Knapp F.F.J., Febbraio M., Beets A.L., Silverstein R.L., Abumrad N.A. (2000). Defective uptake and utilization of long chain fatty acids in muscle and adipose tissues of CD36 knockout mice. J. Biol. Chem..

[49] Yang J., Park K.W., Cho S. (2018). Inhibition of the CD36 receptor reduces visceral fat accumulation and improves insulin resistance in obese mice carrying the BDNF-Val66Met variant. Biol. Chem..

[50] Kennedy D.J., Kuchibhotla S., Westfall K.M., Silverstein R.L., Morton R.E., Febbraio M. (2011). A CD36-dependent pathway enhances macrophage and adipose tissue inflammation and impairs insulin signalling. Cardiovasc. Res..

[51] Hotamisligil G.S., Shargill N.S., Spiegelman B.M. (1993). Adipose expression of tumor necrosis factor-alpha: Direct role in obesity-linked insulin resistance. Science.

[52] Song Z., Chen C., He J., Liu B., Ji W., Wu L., He L. (2022). ASK1-interacting protein 1 acts as a novel predictor of type 2 diabetes. Front. Endocrinol..

[53] Zhai H., Wang D., Wang Y., Gu H., Jv J., Yuan L., Wang C., Chen L. (2024). Kaempferol alleviates adipose tissue inflammation and insulin resistance in *db*/*db* mice by inhibiting the STING/NLRP3 signaling pathway. Endocr. Connect..

[54] Uysal K.T., Wiesbrock S.M., Marino M.W., Hotamisligil G.S. (1997). Protection from obesity-induced insulin resistance in mice lacking TNF-alpha function. Nature.

[55] Kanda H., Tateya S., Tamori Y., Kotani K., Hiasa K., Kitazawa R., Kitazawa S., Miyachi H., Maeda S., Egashira K. (2006). MCP-1 contributes to macrophage infiltration into adipose tissue, insulin resistance, and hepatic steatosis in obesity. J. Clin. Investig..

[56] Tamura Y., Yano M., Kawao N., Okumoto K., Ueshima S., Kaji H., Matsuo O. (2013). Enzamin ameliorates adipose tissue inflammation with impaired adipocytokine expression and insulin resistance in *db*/*db* mice. J. Nutr. Sci..

[57] Bastard J.P., Maachi M., Van Nhieu J.T., Jardel C., Bruckert E., Grimaldi A., Robert J.J., Capeau J., Hainque B. (2002). Adipose tissue IL-6 content correlates with resistance to insulin activation of glucose uptake both in vivo and in vitro. J. Clin. Endocrinol. Metab..

[58] Sabio G., Das M., Mora A., Zhang Z., Jun J.Y., Ko H.J., Barrett T., Kim J.K., Davis R.J. (2008). A stress signaling pathway in adipose tissue regulates hepatic insulin resistance. Science.

[59] Cai D., Yuan M., Frantz D.F., Melendez P.A., Hansen L., Lee J., Shoelson S.E. (2005). Local and systemic insulin resistance resulting from hepatic activation of IKK-beta and NF-kappaB. Nat. Med..

[60] Orland M.J., Permutt M.A. (1987). Quantitative analysis of pancreatic proinsulin mRNA in genetically diabetic (*db*/*db*) mice. Diabetes.

[61] Pretz D., Le Foll C., Rizwan M.Z., Lutz T.A., Tups A. (2021). Hyperleptinemia as a contributing factor for the impairment of glucose intolerance in obesity. FASEB J..

[62] Chen L., Cao H., Huang Q., Xiao J., Teng H. (2022). Absorption, metabolism and bioavailability of flavonoids: A review. Crit. Rev. Food Sci. Nutr..

[63] Baky M.H., Elshahed M., Wessjohann L., Farag M.A. (2022). Interactions between dietary flavonoids and the gut microbiome: A comprehensive review. Br. J. Nutr..

[64] Shimizu R., Shimabayashi H., Moriwaki M. (2006). Enzymatic production of highly soluble myricitrin glycosides using β-galactosidase. Biosci. Biotechnol. Biochem..

[65] Xiang D., Wang C., Wang W., Shi C.Y., Xiong W., Wang M.D., Fang J.G. (2017). Gastrointestinal stability of dihydromyricetin, myricetin, and myricitrin: An in vitro investigation. Int. J. Food Sci. Nutr..

[66] Semwal D.K., Semwal R.B., Combrinck S., Viljoen A. (2016). Myricetin: A dietary molecule with diverse biological activities. Nutrients.

[67] American Institute of Nutrition (1977). Report of the American institute of nutrition ad hoc committee on standards for nutritional studies. J. Nutr..

[68] Bieri J.G. (1980). Second Report of ad hoc committee on standards for nutritional studies. J. Nutr..

[69] Folch J., Lees M., Sloane Stanley G.H. (1957). A simple method for the isolation and purification of total lipids from animal tissues. J. Biol. Chem..

[70] Kim H.J., Lee K.T., Park Y.B., Jeon S.M., Choi M.S. (2008). Dietary docosahexaenoic acid-rich diacylglycerols ameliorate hepatic stea- tosis and alter hepatic gene expressions in C57BL/6J-Lep*ob*/*ob* mice. Mol. Nutr. Food Res..

[71] Davidson A.L., Arion W.J. (1987). Factors underlying significant under- estimations of glucokinase activity in crude liver extracts: Physiological implications of higher cellular activity. Arch. Biochem. Biophys..

[72] Alegre M., Ciudad C.J., Fillat C., Guinovart J.J. (1988). Determination of glucose-6-phosphatase activity using the glucose dehydrogenase-coupled reaction. Anal. Biochem..

[73] Bentle L.A., Lardy H.A. (1976). Interaction of anions and divalent metal ions with phosphoenolpyruvate carboxykinase. J. Biol. Chem..

[74] Nepokroeff C.M., Lakshmanan M.R., Porter J.W. (1975). Fatty acid synthase from rat liver. Methods Enzymol..

[75] Markwell M.A., McGroarty E.J., Bieber L.L., Tolbert N.E. (1973). The subcellular distribution of carnitine acyltransferases in mamma-lian liver and kidney. A new peroxisomal enzyme. J. Biol. Chem..

[76] Lazarow P.B. (1981). Assay of peroxisomal β-oxidation of fatty acids. Methods Enzymol..

[77] Bradford M.M. (1976). A rapid and sensitive method for the quantitation of microgram quantities of protein utilizing the principle of protein-dye binding. Anal. Biochem..

[78] Schmittgen T.D., Livak K.J. (2008). Analyzing real-time PCR data by the comparative CT method. Nat. Protoc..

